# On the mechanism of droplet rolling and spinning in inclined hydrophobic plates in wedge with different wetting states

**DOI:** 10.1038/s41598-021-94523-8

**Published:** 2021-07-23

**Authors:** Bekir Sami Yilbas, Mubarak Yakubu, Abba Abdulhamid Abubakar, Hussain Al-Qahtani, Ahmet Sahin, Abdullah Al-Sharafi

**Affiliations:** 1 Mechanical Engineering Department, King Fahd University of Petroleum and Minerals, Dhahran, 31261 Saudi Arabia; 2 Center of Research Excellence in Renewable Energy (CoRE-RE), KFUPM, Dhahran, 31261 Saudi Arabia; 3 Senior Researcher at K.A. CARE Energy Research & Innovation Center at Dhahran, Dhahran, Saudi Arabia

**Keywords:** Renewable energy, Mechanical engineering

## Abstract

A water droplet rolling and spinning in an inclined hydrophobic wedge with different wetting states of wedge plates is examined pertinent to self-cleaning applications. The droplet motion in the hydrophobic wedge is simulated in 3D space incorporating the experimental data. A high-speed recording system is used to store the motion of droplets in 3D space and a tracker program is utilized to quantify the recorded data in terms of droplet translational, rotational, spinning, and slipping velocities. The predictions of flow velocity in the droplet fluid are compared with those of experimental results. The findings revealed that velocity predictions agree with those of the experimental results. Tangential momentum generated, via droplet adhesion along the three-phase contact line on the hydrophobic plate surfaces, creates the spinning motion on the rolling droplet in the wedge. The flow field generated in the droplet fluid is considerably influenced by the shear rate created at the interface between the droplet fluid and hydrophobic plate surfaces. Besides, droplet wobbling under the influence of gravity contributes to the flow inside the rolling and spinning droplet. The parallel-sided droplet path is resulted for droplet emerging from the wedge over the dusty surface.

## Introduction

Hydrophobic surfaces become favorable in various applications such as medicine^1^, energy harvesting^2^, heat transfer enhancement^3^, and self-cleaning of surfaces^4,5^. Hydrophobic surfaces have unique texture characteristics, which compose of micro/nanopillars and cavities. The trapped air in micro/nano texture gaps creates non-contacting regions on the surface while reducing the pinning of dislodged particles over surfaces. In addition, the trapped air also lowers interfacial resistance between liquid(s) and the textured surface^6^. In general, liquids on smooth surfaces generate low contact angles because of the wetting characteristics of the hydrophilic surfaces. Oppositely, liquid spreading can be minimized via proper texturing of the surfaces. Depending on the texture properties, not only the contact angle of the liquid droplets is improved, but the droplet pinning can be lowered via generating the Lotus effect over the textured surfaces^7^. The lotus effect creates low contact angle hysteresis and the droplet sliding replaces with the rolling under the gravitational influence as the textured surface is inclined. The droplet rolling remains one of the approaches in self-cleaning applications towards mitigating dust from surfaces^8^. Although several parameters affect the droplet rolling, the main influencing parameters are droplet fluid surface tension, interfacial resistance between droplet fluid and surface, droplet size, hydrophobic surface tilting angle, and contact hysteresis. The large size droplets are preferable in self-cleaning applications because of increased dust mitigated area along the droplet path over the dusty surfaces. However, as the droplet volume increases, droplet puddles forming a non-spherical droplet shape on the hydrophobic surface. Hence, the large size droplets geometrically wobble, which can cause fluctuating three-phase-contact length over the inclined hydrophobic surface. This creates a non-uniform droplet path along the dusty surface while causing varying dust mitigated area over the surface, i.e. dust mitigated area appears to have striation-like patterns, which are caused by the wobbling of the rolling droplet along its path. One of the methods of reducing the striations along the droplet path is to create a droplet spinning along its vertical axis during rolling^9^. The spinning of the rolling droplet can be achieved through creating the tangential momentum by a mechanical arrangements^9^. The droplet fluid behavior remains important in spinning and rolling droplets for creating parallel-sided droplet paths over the dust hydrophobic surfaces. Consequently, investigation of droplet fluid behavior becomes essential during droplet spinning and rolling pertinent to effective dust mitigation from the hydrophobic surfaces.

The surface wettability and droplet fluid penetration into the hydrophobized texture play an important role in droplet mobility. The large texture gaps allow droplet fluid penetration into the hydrophobized surface while increasing droplet pinning, which becomes apparent for large volume water droplets. The critical condition for droplet penetration into surface texture demonstrates a parabolic relation between the droplet and the gap sizes in the texture^10^. In some cases, the droplet fluid penetration can result in liquid spreading while affecting the droplet shape on the surface. This causes droplet instability in terms of droplet localized disintegration from the original state and oscillation^10^. The surface texture characteristics are also related to droplet pinning, slip, and rolling dynamics on the hydrophobic surfaces. In some cases, the texture demonstrates para-hydrophobic characteristics, which can cause droplet fluid infiltration towards the textured surface and droplet sticking/sliding becomes unavoidable^11^. The slipping of the rolling droplet over the hydrophobic surface can also be resulted because of the interfacial forces due to the molecular interactions between the droplet liquid and the solid surface. In this case, the caterpillar-like rotational flow over the sliding droplet interface is created^12^. Moreover, the rolling droplet on the hydrophobic surfaces finds applications in the self-cleaning of surfaces. Due to low tension at the interfacial between the droplet fluid and the hydrophobic surface, water droplets roll rather than slipping over the surface. As the interfacial resistance increases, droplet rolling replaces with slipping motion^13^. Nevertheless, in both cases (rolling and slipping) droplet fluids can peak-up micro-size particles, such as dust, from the surface. However, to improve the mode of droplet rolling, the Lotus effect needs to be created over the hydrophobic surface^14^. The Lotus effect can be realized through creating nano-size whiskers-like texture structures over the surfaces. Controlling whiskers' size and their uniform distribution over the surface remains challenging. With arbitrarily distributed whiskers-like textures over the surface can cause droplet rolling motion in random directions over the surface. This creates irregular dust mitigated patterns with non-uniform widths over the dusty hydrophobic surfaces. On the other hand, surface coating is one of the alternative methods creating a superhydrophobic wetting state on the surfaces for droplet rolling in self-cleaning applications. However, achieving optically transparent superhydrophobic coated surfaces becomes one of the necessary conditions, particularly, for self-cleaning application of photovoltaic surfaces. Although introducing multi-layer coatings improves the droplet mobility on the superhydrophobic surfaces^15^, such coating attenuates and scatters the incident radiation. The stability of the superhydrophobic coating in practical applications remains important even though high optical transparency of the coating is achieved such as depositing fluoropolymer brush grafted silica nanoparticles on surfaces^16^. An approach introducing coatings exhibiting both self-cleaning and self-healing properties, such as poly(urea-urethane) (PU) coatings, is one of the recent developments in self-cleaning applications^17^; nevertheless, the optical transparency of such coatings needs further investigations for adopting the coating technology in self-cleaning applications of photovoltaic panel surfaces.

Large width, straight, and parallel-sided path of the rolling droplets become crucial in maintaining the effective ways of self-cleaning of dusty hydrophobic surfaces. A large size droplet path width can be achieved by rolling large size droplets on dusty hydrophobic surfaces. However, increasing droplet size enhances droplet wobbling over the surface because of the gravitational effect^18^. Wobbling of large size droplets can create striations-like edges along the droplet path. This gives rise none-uniform path of particles removed from the hydrophobic surfaces while degrading the efficient self-cleaning of surfaces. However, this disadvantage can be eliminated through introducing spinning of the rolling droplets over the hydrophobic surfaces. In this case, the droplet path yields uniform and self-cleaning of the hydrophobic surface becomes efficient. On the other hand, to enhance the droplet spinning, an imbalance of forces acting around the tangential plane (normal to the rolling plane) of the droplet needs to be created. The imbalance tangential forces can be created in an inclined hydrophobic wedge structure with two flat hydrophobic surfaces having different wetting states (contact angles). This arrangement gives rise to the lateral imbalance of the pinning forces, which can cause the spinning of the rolling droplet. As the rolling and spinning droplet moves over the hydrophobic surface, it results in a straight line of motion with the path having almost parallel-sided edges. Moreover, the flow behavior in the droplet fluid influences the spinning and rolling behavior of the droplet on the hydrophobic surfaces. Although droplet rolling over the inclined hydrophobic surfaces ^8^ and flow field in a sessile droplet under thermal loading ^19,20^ have been investigated previously, the flow behavior inside the spinning and rolling droplet fluid giving rise to a straight projectile motion was left for future study. Consequently, in the present study, rolling and spinning droplet motion within the inclined wedge structure having different hydrophobic states of wedge plates is considered. The flow field developed inside the droplet is simulated in three-dimensional space via adopting the experimental conditions. A high-speed camera system is used to record the three-dimensional droplet motion within the hydrophobic wedge as well as over the hydrophobic plain surface. A tracker program is used to quantify the rolling and spinning velocities of the droplet in hydrophobic wedge and on the plain hydrophobic surface. The flow predictions are compared with those of the experimental findings. Moreover, the path of rolling and spinning droplets is evaluated using the dusty plain hydrophobic surface. The rolling droplet path on plain hydrophobic surface (without spinning) is also assessed and findings are compared with its counterpart obtained for rolling and spinning droplets. In addition, the rolling and spinning velocities of the droplet obtained from analytical solutions^9^ are compared with the 3D predictions and the experimental data.

## Numerical and validation study

**Figure 1:**
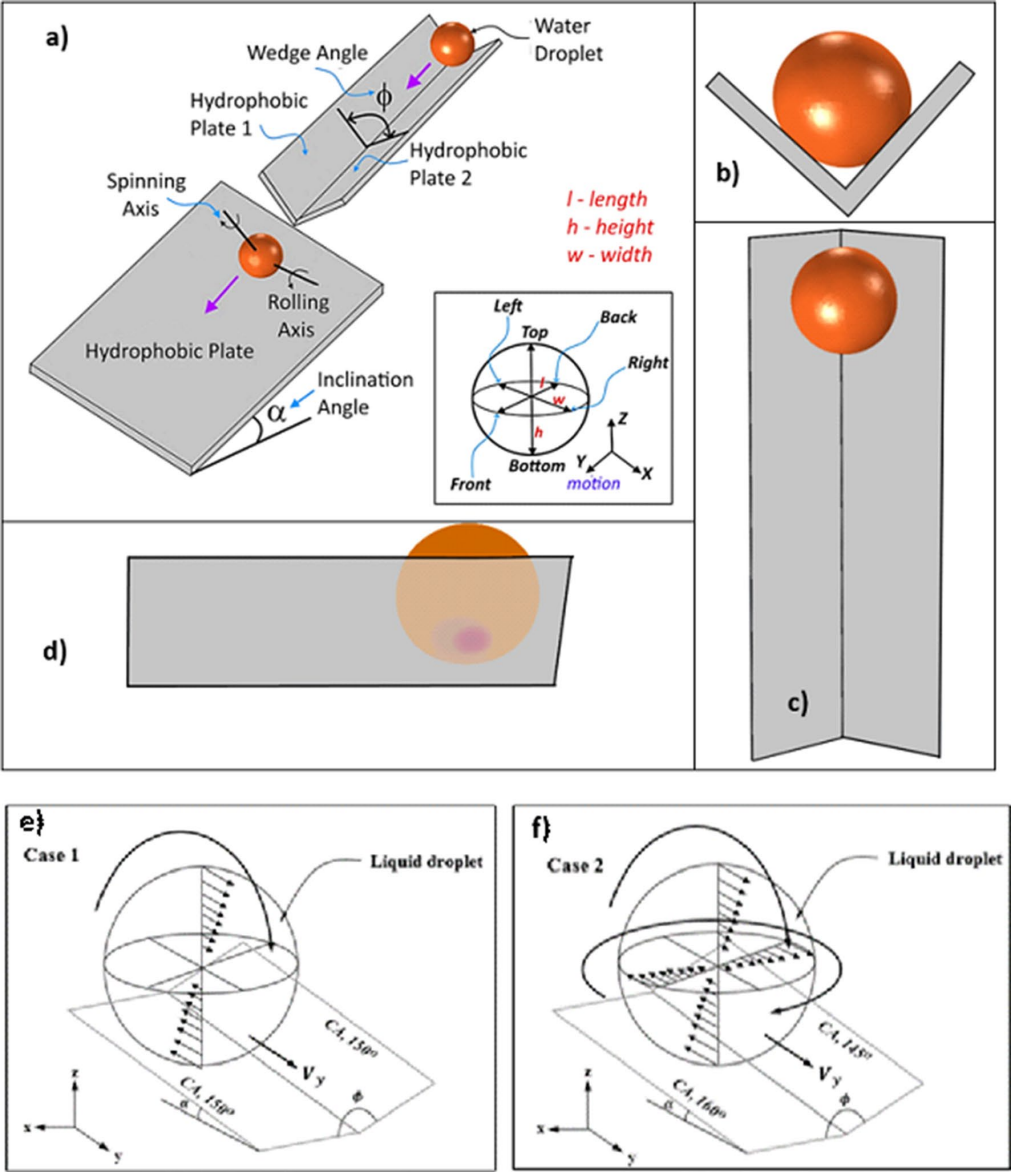
Schematic view of inclined wedge fixture: (**a**) design perspective view of fixture, (**b**) front view of fixture and droplet, c) top view of fixture and droplet, (**d**) side view of fixture and droplet, (**e**,**f**) cases considered in numerical simulations.

Simulation of the flow field inside spinning and rolling droplet in the inclined hydrophobic wedge structure (Fig. 1) requires the coupling of Navier Stokes’s equation and level-set model. The conservation equations for incompressible laminar and viscous flow are considered when formulating the droplet spinning and rolling behavior in the inclined hydrophobic wedge. The continuity equation for the conservation of mass can be stated as:

1 $$\nabla \cdot \bar{\nu } = 0$$

here $\bar{\nu }$ is velocity vector.

The momentum equation can be expressed as:

2 $$\rho \frac{{\partial \bar{\nu }}}{{\partial t}} + \rho (\bar{\nu } \cdot \nabla )\bar{\nu } = - \nabla \cdot \left[ { - p\bar{I} + \mu \left( {\nabla \bar{\nu } + \left( {\nabla \bar{\nu }} \right)^{T} } \right)} \right] - \rho \bar{g} - \sigma k\delta \bar{n} - F_{{fr}}$$

where: $\rho$ is density, $\bar{g}$ is acceleration vector due to gravity, $p$ is gauge pressure, $\sigma$ is surface tension, $k = \nabla \cdot \frac{{\bar{n}}}{{\left| {\bar{n}} \right|}}$ is curvature, $\overline{n }$ is a unit vector normal to the interface, $\delta$ is Dirac Delta function, ${F}_{fr}$ is friction source term, and $t$ is time.

The conservative level set equation for two-phase flow of droplet within the hydrophobic wedge structure and surrounding air can be expressed as^21^:

3 $$\frac{{\partial \phi }}{{\partial t}} + \nabla (\bar{\nu }\phi ) = \gamma \cdot \nabla \cdot \left( {\varepsilon _{{ls}} \nabla \phi - \phi (1 - \phi )\frac{{\nabla \phi }}{{\left| {\nabla \phi } \right|}}} \right)$$

where: $\phi$ is the level-set function, $\gamma$ is re-initialization parameter, and ${\varepsilon }_{ls}$ is the parameter that controls interface thickness.

Incorporating the level-set function, the droplet properties such as density and viscosity of the fluids can be scaled according to the mixture rule, i.e.: $\rho ={\rho }_{2}+({\rho }_{1}-{\rho }_{2})\phi$.

The energy equation is:

4 $$\rho {C}_{p}\frac{\partial T}{\partial t}+\rho {C}_{p}\overline{u }\cdot \nabla T=\nabla \cdot \left(k\nabla T\right)$$

Here: $T$ is temperature, ${C}_{p}$ is effective specific heat, and *k* is effective thermal conductivity.

Droplet surface evaporation into the air ambient can be included via utilizing diffusion-convection analogy, which can be written as^22^:

5 $$\frac{\partial {C}_{v}}{\partial t}+\nabla \bullet \left(-{D}_{v}\nabla {C}_{v}\right)+{\overline{u} }_{f}\bullet \nabla {C}_{v}=0$$

Here: *C*_*v*_ is the specific heat at constant volume, ${\overline{u} }_{f}$ being airflow velocity, and *D*_*v*_ is diffusion coefficient (m^2^ s^−1^). The interfacial mass-flux at droplet fluid and air contact surface can be obtained through the adoption of the mass-conservation terms, which are^23,24^:

6 $$\left\{\begin{array}{l}J={\rho }_{l} \left(\overline{{u }_{l}}\bullet {\overline{n} }_{int}-{\overline{\upsilon }}_{int}\right) \\ J={\rho }_{v}\left({\overline{u} }_{v}\bullet {\overline{n} }_{int}-{\overline{\upsilon }}_{int}\right)-{D}_{v}\nabla {\rho }_{v}\bullet {\overline{n} }_{int}=0\end{array}\right.$$

Here: ${\overline{\upsilon }}_{int}$ represents interfacial velocity, ${\overline{n} }_{int}$ is outward unit vector being normal to droplet fluid-air interface, vapor phase is specified by *v* and liquid phase is by *l*. Therefore, change of velocity across the interface can be written as^24^:

7 $$\left({\overline{u} }_{l}-{\overline{u} }_{g}\right)\bullet \overline{n }=\mathrm{J}\left(\frac{1}{{\rho }_{l}}-\frac{1}{{\rho }_{v}}\right)-{D}_{v}\frac{\nabla {\rho }_{v}\bullet \overline{n}}{{\rho }_{v}}$$

After rearranging Eqs. (6) with (7), droplet surface velocity (shrinking surface velocity) can be reduced to:

8 $${\overline{\upsilon }}_{l}={\overline{u} }_{l}\bullet \overline{n }-\frac{\mathrm{J}}{{\rho }_{l}}$$

Moreover, surface stresses occur due to change of interfacial velocities in gas and liquid sides. Hence, Marangoni shear can be incorporated at droplet interface and it can be expressed as:

9 $${\left({\overline{n} }_{int}\bullet \tau \right)}_{v}={\left({\overline{n} }_{int}\bullet \tau \right)}_{l}{F}_{st}$$

Here, *τ* is the stress tensor (N m^−2^) and ${F}_{st}$ is the surface tension force.

The surface tension force can be written as:

10 $${F}_{st}=\gamma \left({\nabla }_{\mathrm{int}}\bullet n\right)n-{\nabla }_{\mathrm{int}}\gamma$$

Here, $\gamma \left({\nabla }_{\mathrm{int}}\bullet n\right)n$ represents interfacial force per unit area, $\gamma$ is droplet fluid surface tension, and ${\nabla }_{\mathrm{int}}{\gamma }_{\mathrm{1,2}}={\gamma }^{^{\prime}}{\nabla }_{\mathrm{int}}T$ is corresponding to tangential stress due to evaporation, ${\gamma }^{^{\prime}}$ is the gradient of interfacial-surface tension (N m^−1^ K^−1^)), ${\nabla }_{\mathrm{int}}T$ is temperature gradient at the interface, and index $int$ demonstrates interface.

### Initial and boundary conditions

**Figure 2:**
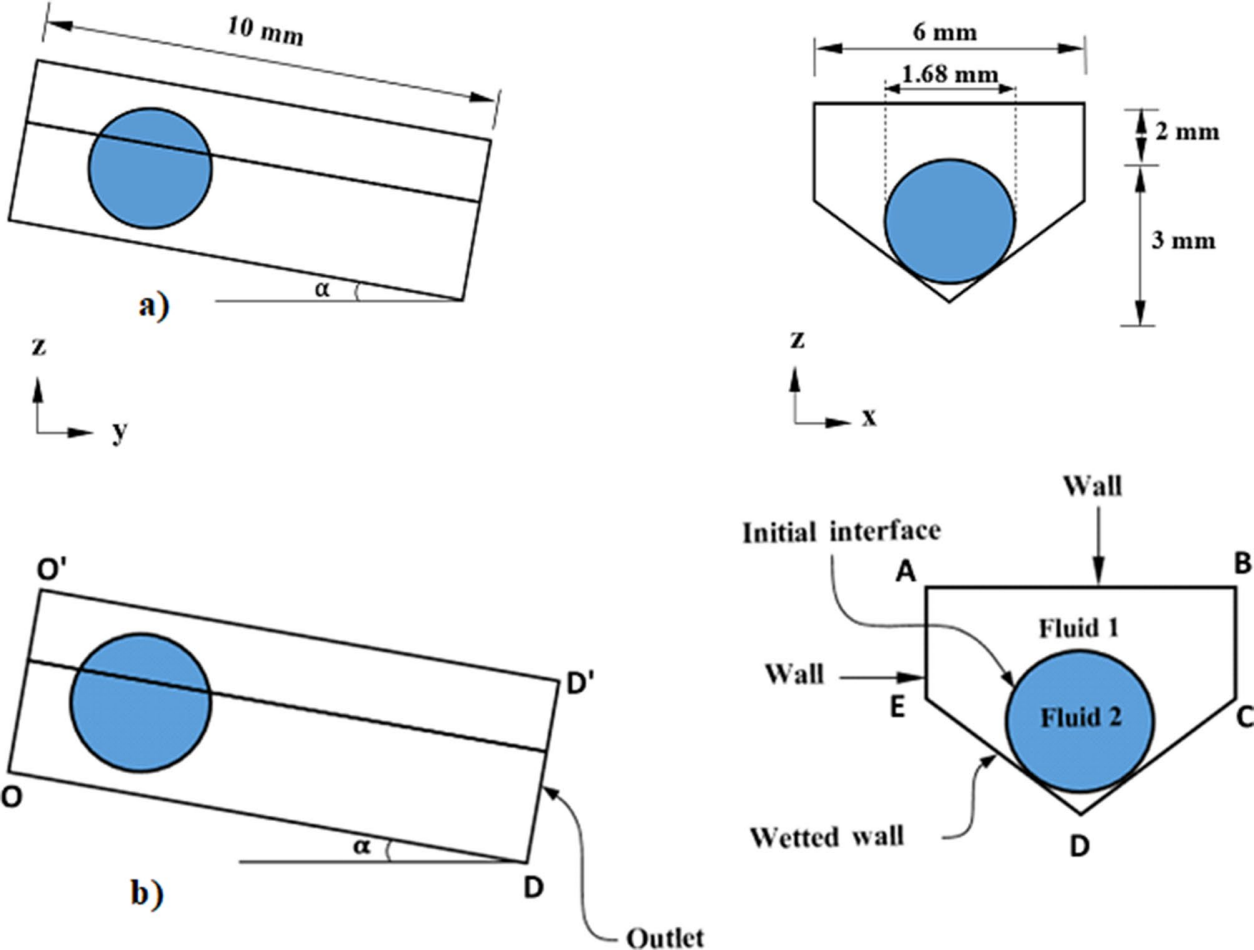
(**a**) Geometric configuration of spinning and rolling droplet in the wedge fixture, (**b**) Boundaries conditions adopted in numerical simulations.

The schematic view of the droplet and the fixture used in experiments and simulations is demonstrated in Fig. 1a and cases considered in the simulations are depicted in Fig. 1e,f. The geometrical arrangements of the droplet in the wedge are shown in Fig. 2a,b. Two cases are incorporated in the simulations. In the first case (case (a) in Fig. 1e), the water droplet (20μL) is considered to roll down over 10° inclined wedged fixture, which is formed by two surfaces having same hydrophobic states, i.e. both surfaces have the contact angle of 150°. In the second case (case (b) in Fig. 1f), all parameters are kept similar to case (a); however, the contact angle of the two surfaces are changed to 145° and 160° (left plate surface has the contact angle of 145° and right plate surface has 160° in the wedge). Moreover, for cases (1e) and (1f), the water droplet is considered to be initially at rest and, later, it moves down under the gravity in the inclined wedged fixture. Hence, the initial conditions utilized for the simulations can be expressed as follows:

11 $$\bar{\nu }\left( {x,y,z,0} \right) = 0\;m/s\;for\;cases\;\left( a \right)\;and\;(b)$$

12 $$T\left( {x,y,z,0} \right) = 300\;K\;for\;air\;and\;303\;K\;for\;droplet$$

13 $$p(x,y,z,0)=0$$

14 $$(\phi (x,y,z,0) = \left\{ {\begin{array}{ll} 1 & {{\text{for}}\;{\text{droplet}}} \\ {0.5} & {{\text{for}}\;{\text{interface}}} \\ 0 & {{\text{for}}\;{\text{air}}} \\ \end{array} } \right.$$

For the boundary conditions, in line with the level set formulation, the interface between the droplet and air is considered to move freely within the computational domain (Fig. 2a,b) with Marangoni condition imposed. Two substrate surfaces (CD and DE. Figure 2b) are considered to have the hydrophobic walls in which the contact angles between the droplet, air, and wall are defined and droplet slipping is also allowed at such boundary. Boundaries EABC (Fig. 2b) are considered to be hydrophobic walls having slipping boundary conditions. Boundary OO’ in Fig. 2b is considered to be a free surface, while the pressure outlet is defined at boundary DD’. Thermal insulation is applied at exterior exposed surfaces of domain. Thus, the boundary conditions used can be expressed as:

15 $$\left. {p(x,y,z,t)} \right|_{{DD^{\prime}}} {\text{ }} = {\text{ }}0\;\;\;({\text{pressure}}\;{\text{outlet}}\;{\text{at}}\;DD^{\prime},\;{\text{Figure}}\;2{\text{b}}))$$

16 $$\left. {\bar{u}(x,y,z,t)} \right|_{{at{\text{ EABC}}}} \cdot \bar{n}_{{wall}} = 0\;\;\;({\text{slip}}\;{\text{at}}\;{\text{the}}\;{\text{walls}}\;{\text{EABC}},\;{\text{Figure}}2{\text{b}})$$

17 $$\left. {\bar{u}(x,y,z,t)} \right|_{{at{\text{ CDE}}}} \cdot \bar{n}_{{wall}} = 0\;\;\;\;({\text{slip}}\;{\text{at}}\;{\text{wetted}}\;{\text{wall}}\;{\text{at}}\;{\text{CDE}},\;{\text{Figure}}2{\text{b}})$$

18 $$F_{{fr}} = \gamma (\bar{n}_{{wall}} - \left( {\bar{n}cos\theta _{d} \frac{\mu }{\beta }\left. {\bar{u}\left( {x,y,z,t} \right)} \right|} \right)\left| {at{\text{ CDE}}_{{\text{int} }} } \right.\;\;\;({\text{frictional}}\;{\text{force}}\;{\text{at}}\;{\text{wetted}}\;{\text{walls}}\;{\text{CDE}},\;{\text{Figure}}2{\text{b}})$$

where: $\gamma$ is the surface tension of the fluid, $\Gamma$ is boundary path, ${n}_{wall}$ is a unit vector normal to the surface, ${n}_{int}$ is a unit vector normal to the contact line, ${\theta }_{d}$ is the dynamic contact angle, $\delta$ is Dirac-Delta function, $\beta$ is slip length, and $t$ is time. The dynamic contact angle is implemented based on the quasi-dynamic model provided in previous studies ^25,26^.

### Numerical implementation

**Figure 3:**
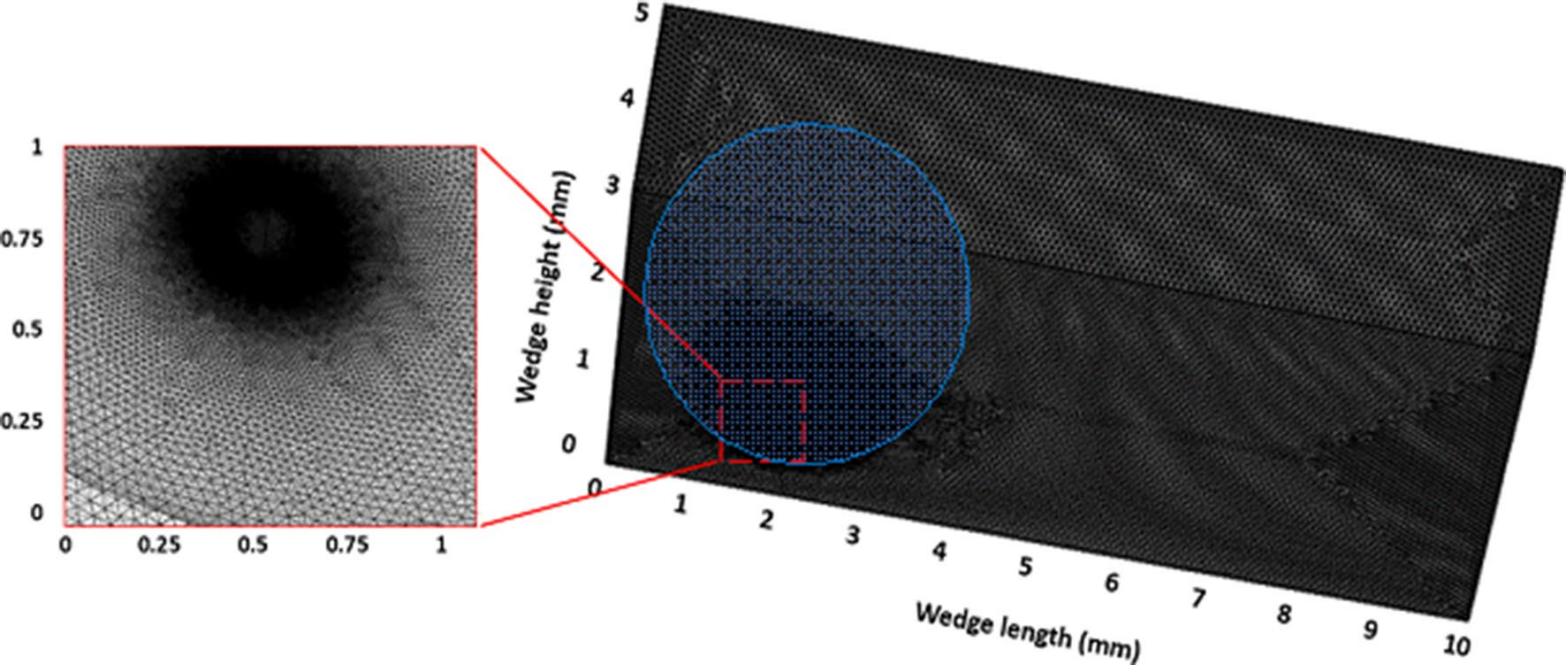
Grid used in simulations. Finer and dense meshes were located across water–air and water-hydrophobic surface interfaces.

**Figure 4:**
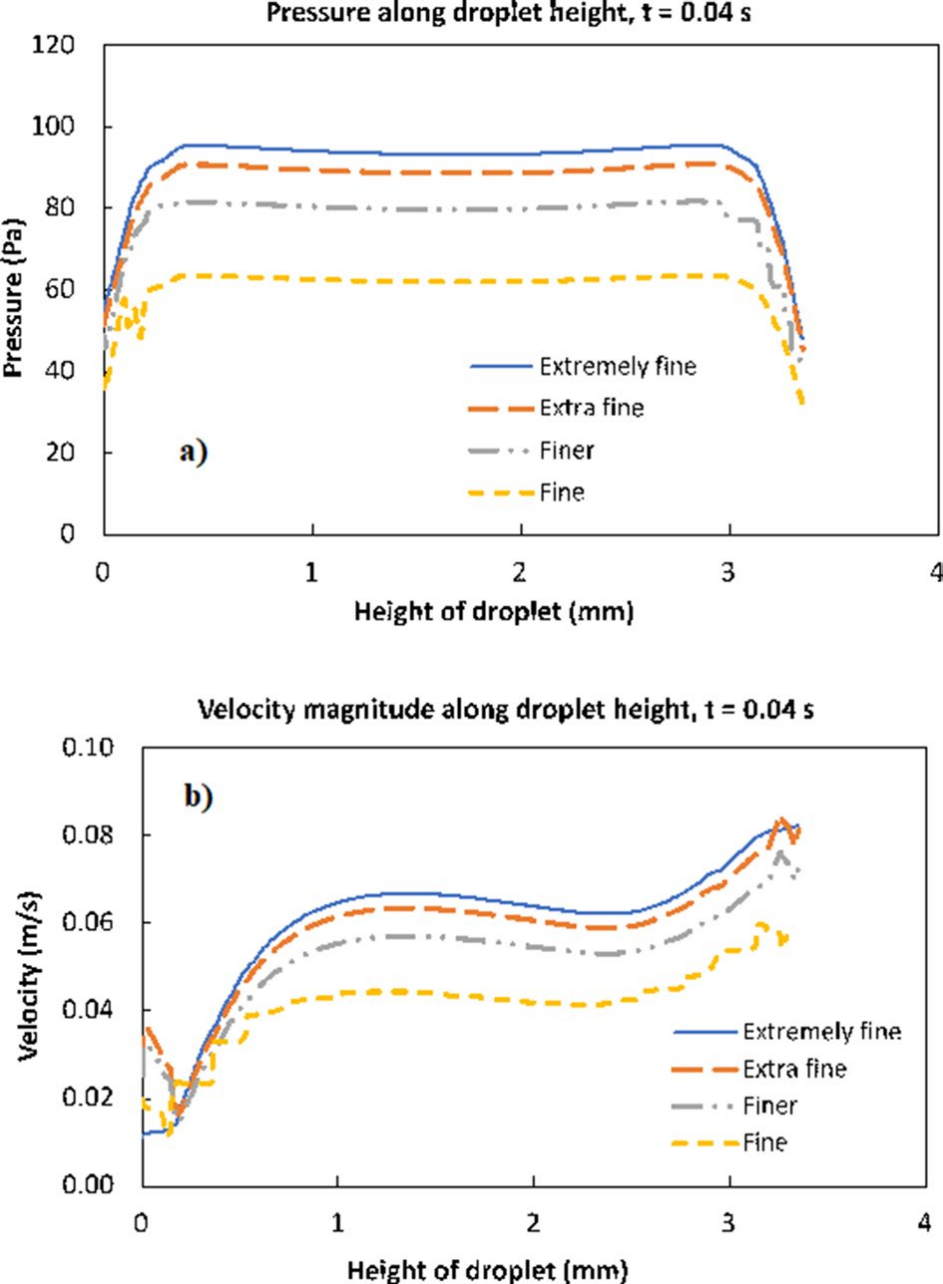
Grid independence solutions for (**a**) velocity magnitude and (**b**) pressure variation along droplet height from bottom to top at 0.04 s. Fine, finer, extra fine and extremely fine mesh has 161,511, 568,536, 2,175,389, and 9,471,536 tetrahedral elements, respectively.

COMSOL multi-physics code ^27^ is used to model the rolling and spinning of the droplet in the wedge with different hydrophobic states while incorporating the equations (Eqs. (1) to (17)) as well as initial and boundary conditions. The numerical model is built for 3D simulations. The numerical accuracy of the scheme is mainly dependent on the time step size, mesh size, and level-set parameters. In the simulations, the time step as small as 10^−8^ s is used for the numerical solution ensuring the convergence of the time derivatives. The second-order Euler backward difference scheme is incorporated discretizing the time derivatives in the flow and energy equations. For the level-set approach, the droplet interface is treated implicitly such that the numerical convergence is achieved with a regular mesh of sufficient density (shown in Fig. 3). In addition, care is taken towards setting the level-set parameters (${\gamma }_{r}$ and ${\varepsilon }_{ls}$) such that the movement of the droplet can be effectively captured within the fixed meshes. Based on the initial simulation tests and the best practice, the re-initialization parameter (${\gamma }_{r}$) is set to be equal to the maximum velocity of flow while the interface thickness (${\varepsilon }_{ls}$) is considered as half of the maximum element (edge) length. As demonstrated in Fig. 4, the grid-independent solution test results are obtained for pressure and velocity distribution along the droplet height in the vertical direction. Fine, finer, extra fine, and extremely fine mesh have 161,511, 568,536, 2,175,389, and 9,471,536 tetrahedral elements respectively. In the simulations, extra-fine meshes are used. It is worth noting that the typical simulation period (run time) for one case adopting the extra fine mesh (using 20 core and 128 RAM high-performance computer) is 27 days of continuous computation time. Furthermore, mass is conserved during the analysis by ensuring that the change in mass during the rolling and spinning of the droplet is negligibly small (< 10^−8^ g).

### Validation

**Figure 5:**
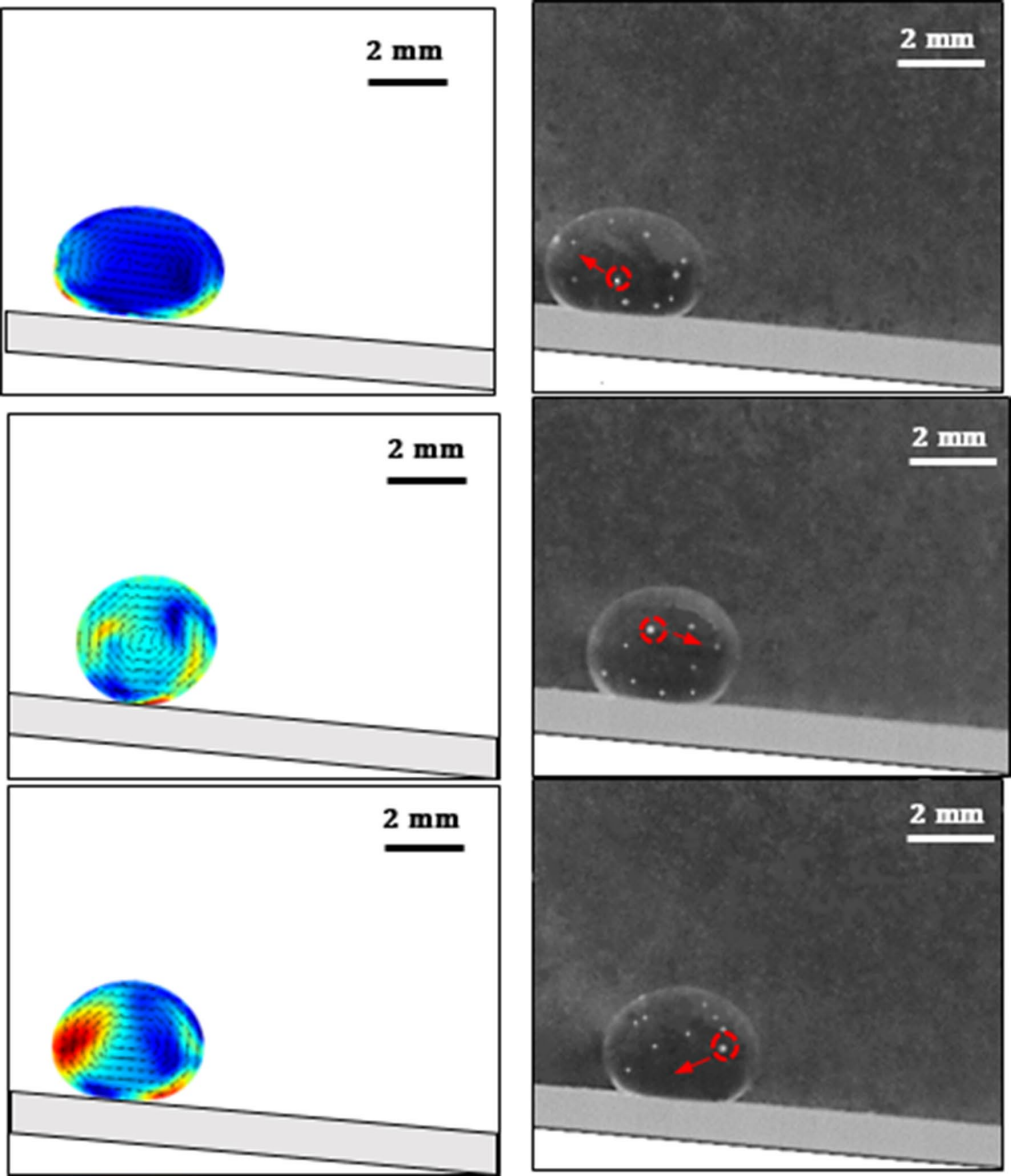
Velocity field developed inside droplet fluid: (a) numerical predictions, (b) high-speed images of droplet and silicon particles. Particles trajectories are shown in the figure.

The validation study is conducted to compare the velocity predictions inside the droplet with the experimental values. Hence, further experiments are realized measuring the velocity inside the spinning and rolling droplet in the wedge. The droplet fluid (distilled water) is mixed with hollow silica particles by 0.5% (volume) and the mixture is shaken ultrasonically ensuring the uniform mixture. Hollow silica particles have a density of 1100 kg/m^3^ and a size of about 20 μm. The mixture liquid is used to deposit a droplet of 20 μL in the wedge fixture having different hydrophobic states (145° and 160°). A high-speed recording facility is used to record the hollow silica particles in the plane of the laser beam shining through the droplet. It is worth mentioning that a beam expander and cylindrical lens are used to expand the laser beam passing through the symmetry plane of the droplet and the hollow silica particles in this plane are monitored with a high-speed camera facility. A tracker program is used to quantify the velocities of the silica particles recorded by the high-speed system. The spherical surface of the droplet fluid can distort the images of the particles monitored. The correction is introduced towards minimizing the influence of the droplet spherical geometry on the optical images while adopting the method used in the early work^28^. Table 1 gives the velocity data obtained from the experiments and the predictions corresponding to the same locations inside the droplet while Fig. 5 shows the images of the droplet and the hollow glasses at different time frames. Some small differences in measured and predicted velocities are observed, which can be related to the experimental and round of errors in the simulations. Nevertheless, the differences are small. The experiments are cycled 12 times to estimate the experimental errors. It is found that the experimental error is about 3%. It should be noted that the standard method is used to assess the error ($\sigma$), i.e. $\sigma =\frac{\sqrt{\frac{\sum_{i=1}^{n}{({x}_{i}-\overline{x })}^{2}}{n-1}}}{\sqrt{n}}$*,* where *n* represents measurement repeats *x*_*i*_ corresponds to the hollow silica particle velocity, and $\overline{x }$ being the mean value of the velocities. Moreover, for the assessment of the velocity of hollow silica particles, the Stokes number ($Stk = \frac{{t}_{o}{u}_{o}}{{l}_{o}}$, where *t*_*o*_ is the relaxation time of the hollow silica particles, *u*_*o*_ is the flow velocity, and *l*_*o*_ is the characteristics size of the particles) is considered. The Stokes number (Stk) of in the order of 0.015 is evaluated, which is much less than unity. Hence, the hollow particles follow the streamlines in the droplet fluid.

**Table 1:** Flow velocities predicted, and obtained from high-speed data and tracker program inside droplet.

Particle #	X (mm)	Y (mm)	Experiment V (m/s)	Simulation V (m/s)
1	1.273456	0.4675	0.0008	0.0008
2	1.058540	0.7434	0.0115	0.0017
3	0.843624	1.0193	0.0207	0.0177
4	0.628708	1.2952	0.0307	0.0396
5	0.413792	1.5711	0.0343	0.0401
6	0.198876	1.8470	0.0340	0.0269
7	− 0.01604	2.1229	0.0332	0.0252
8	− 0.230956	2.3988	0.0345	0.0392
9	− 0.445872	2.0129	0.0387	0.0482
10	− 0.660788	1.6270	0.0455	0.0438
11	− 0.875704	1.2411	0.0489	0.0389
12	− 1.09062	0.8552	0.0521	0.0508
13	− 0.855704	0.4693	0.0539	0.0635
14	− 0.620788	0.0834	0.0528	0.0571
15	− 0.385872	-0.3025	0.0528	0.0446
16	− 0.150956	-0.6884	0.0546	0.0478

Droplet volume is 20 µL, inclination angle is α = 10°, wedge angle is Φ = 90°.

## Experimental

A fixture was designed and built to hold the two hydrophobized glasses forming the inclined wedge (Fig. 1a). The glass plates (30 × 70 × 1 mm^3^, width, length, and thickness) were coated with functionalized silica nano-particles to hydrophobized the surfaces with different wetting states. The silica nanoparticles were prepared and the coating was realized using the technique presented in the early work^8,29^. The contact angle of the hydrophobized surfaces was evaluated using a Goniometer (Kyowa, model DM 501). During the contact angle measurements, a high-drop shape analysis was utilized ^30^. Scanning electron (JEOL 6460 SEM and atomic force (AFM/SPM) microscopes were used to characterize the texture of the hydrophobized surfaces. A high-speed recording facility (SpeedSense 9040, Dantec Dynamic) was used to monitor the droplet motion. The recording facility was operated at 5,000 frames-per-second (fps) and the recorded data had 14 µm × 14 µm pixel sizes and 1280 × 800 resolution. The tracker program was used to quantify the droplet motion in terms of rolling/spinning velocities in the tilted wedge. The uncertainty analysis was conducted to evaluate the uncertainty involved with the high-speed recorded data. In this case, the experiments were cycled 12 times to ensure the experimental repeatability of the data within 3% error. Once the level of confidence of 97% was satisfied with the data recorded, the uncertainty (*σ*_*u*_) was carried out using^31^:

19 $${\sigma }_{u}=\sqrt{{\int }_{{x}_{o}}^{{x}_{n}}{\left(x-{\mu }_{e}\right)}^{2}p\left(x\right)dx}$$

where, *µ*_*e*_ being the mean of *x* (data extracted for the droplet velocity), *n* represents the number of extracted data in the data-set, and *p*(*x*) being the probability function. The value of *p*(*x*) was evaluated over the correlation-plane, and *p*(*x*) was fit into a Gaussian distribution obtaining the diameter. The uncertainty was, later, determined through the Gaussian-fit. *p*(*x*) function resulted was normalized with the number of pixels associated with the cross-correlation peaks. The bias error was evaluated as 0.02 pixels, which were based on the sizing of the extremely small peaks within the *p*(*x*) function. Hence, the uncertainty was estimated at 3.1%.

## Results and discussion

The rolling/spinning of the droplet in inclined hydrophobic wedge having different wetting states of hydrophobic surfaces is examined. The flow field developed inside the droplet is simulated in three-dimensional space. The spinning and rolling droplet path on the dusty hydrophobic surface is monitored to evaluate the geometric features of the path in terms of straightness and parallel-sided edges.

### Hydrophobized wedge surfaces and water droplet

**Figure 6:**
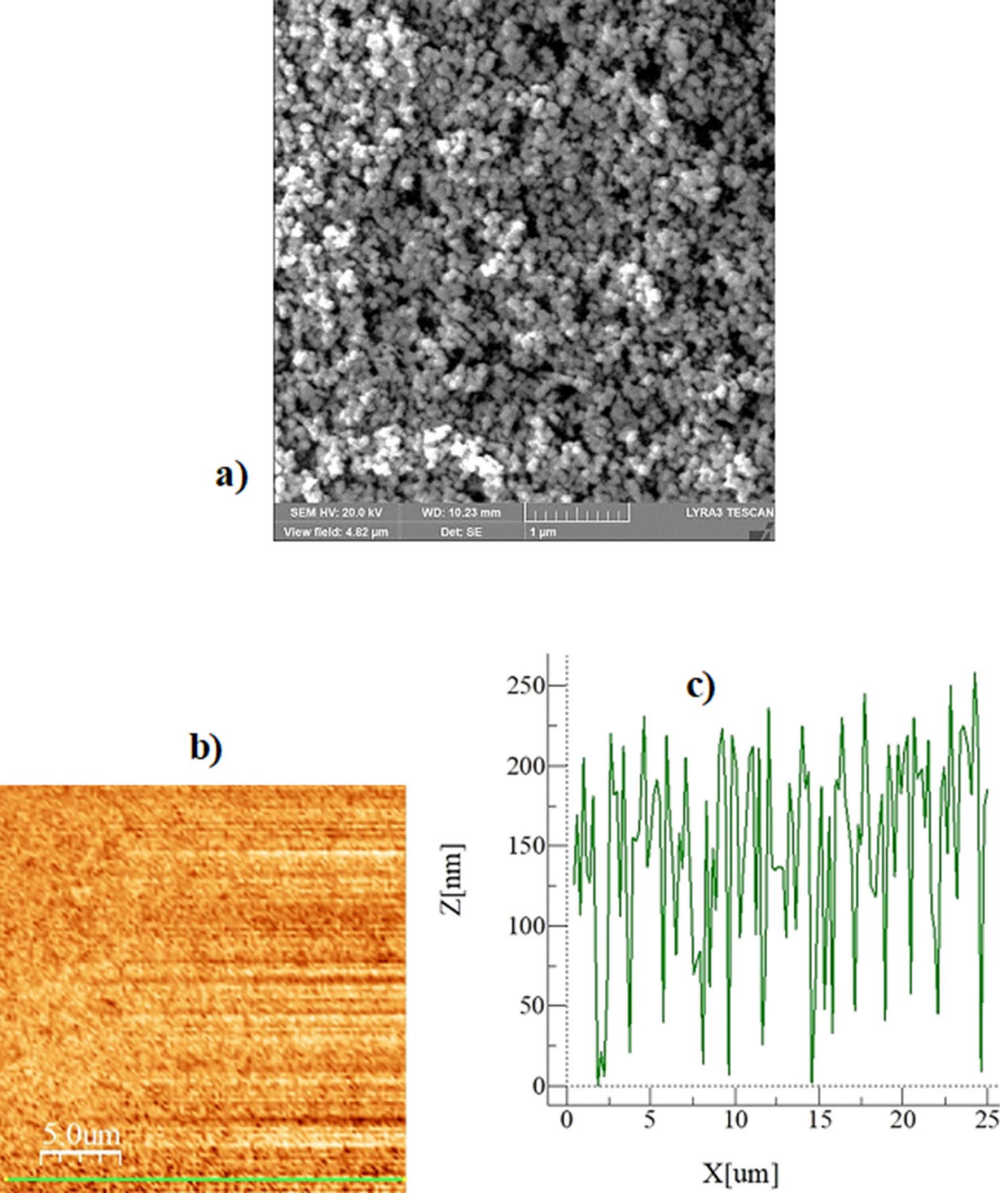
(**a**) SEM micrograph of coated surface, (**b**) AFM image of coated surface, and (**c**) line-scan of coated surface.

**Figure 7:**
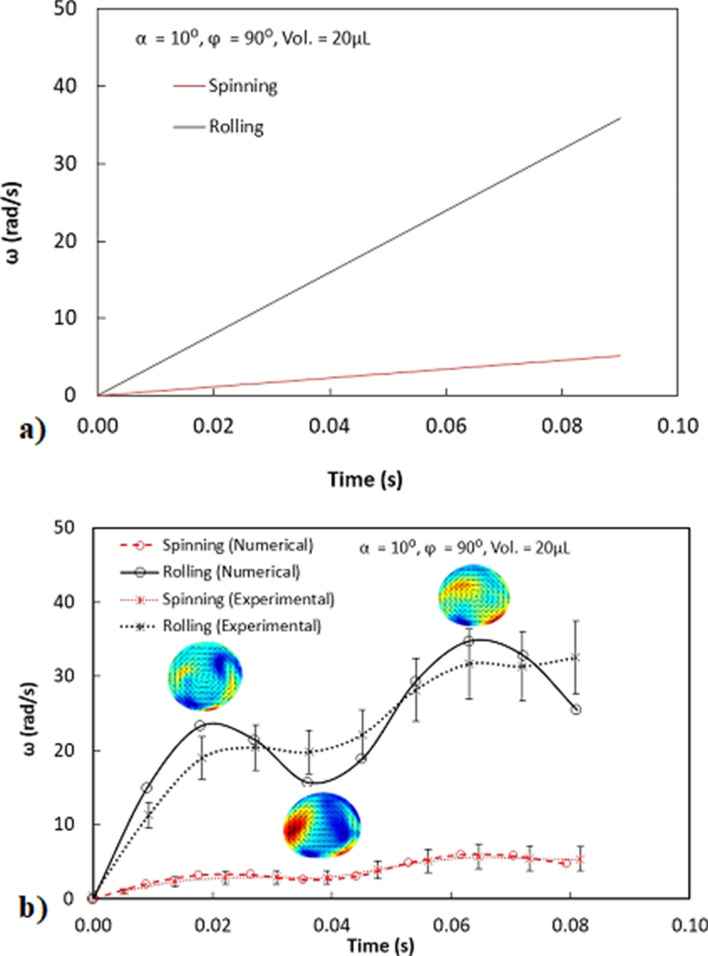
(**a**) Temporal variation of rolling and spinning velocities of droplet in hydrophobic inclined wedge obtained from the analytical formulation ^9^, (**b**) Temporal variation of rolling and spinning velocities of droplet in hydrophobic inclined wedge obtained from simulation and experiments.

Figure 6a shows SEM micro-graph of silica nano-particles coated glass surface while Fig. 6b,c depict the AFM micro-graph and line scan over the coated surface. Nano-particles have almost uniform sizes (about 30 nm) and completely cover the coated surface. A local cluster of the particles appears on the surface, which has the sub-micron cavity-like formations over the coating surface. The local agglomeration of particles is, mainly, resulted from side-reactions of Silone during condensing over the nanoparticle surfaces while causing the grafting of the surface^29^. In addition, the condensing monomers can grow at a faster rate than the nucleation rate while causing agglomeration^29^. The plate surfaces are designed to have different hydrophobic states in the wedge. The contact angle measured for the surface one (right hand side in the wedge) is 160° ± 2° and hysteresis are of 5° ± 1° The contact angle of the second surface (left hand side of the wedge) is 145° ± 2° and hysteresis is of 5° ± 1°. Hence, two different hydrophobic states are achieved according to two different surfaces. The peaks of the agglomerated particles for each surface are apparent from the line scan of AFM (Fig. 6c). The height of the peaks corresponding to the relatively low contact angle of the surface is lower than that of the high contact angle surface. In addition, the texture shows slightly different peak distributions over both surfaces. Nevertheless, the textures of both surfaces result in a hydrophobic state with different contact angles. The average roughness of the surface is about 115 nm for both surfaces. The formulation of droplet rolling is given in early work^9^. The rotational velocity of the droplet increases as the inclination angle of the wedge from the horizontal plane (*α*) increases; however, increasing the wedge angle (*ϕ,* which is the angle between two plane hydrophobic surfaces in the wedge) slows the velocity of the rolling droplet in the wedge^9^. The difference between the retention force on each surface creates a tangential momentum during the droplet rolling. The tangential momentum initiates the spinning of the droplet. Increasing inclination angle of the fixture, in which the droplet rolls and spins, results in increased droplet linear acceleration^9^. In order to achieve the droplet rolling and spinning in the wedge, the wedge fixture geometric angles (*a* and *ϕ*), droplet size (volume and hydraulic radius) must be set accordingly. The detailed analysis is refereed to the supplement (S1 and S2). Figure 7a shows velocity of rolling/spinning droplet with time resulted from the analytical solutions for 20 μL droplet with wedge angle of 90° and inclination angle of the wedge being 10°. The droplet rolling angular velocity over the spinning angular velocity is about 5.5 at the end of 0.08 s of 20 μL droplet motion in the wedge with the wedge angle of 90° and wedge inclination angle of 10°. The ratio shows that the droplet spinning velocity is considerably smaller than the rolling speed. This also demonstrates that the momentum created for the droplet rolling is considerably larger than that of the tangential momentum because of the droplet retention forces generated over the three-phase contact length of the droplet on the hydrophobic wedge surfaces.

### Dynamic and flow analyses for rolling and spinning droplet

**Figure 8:**
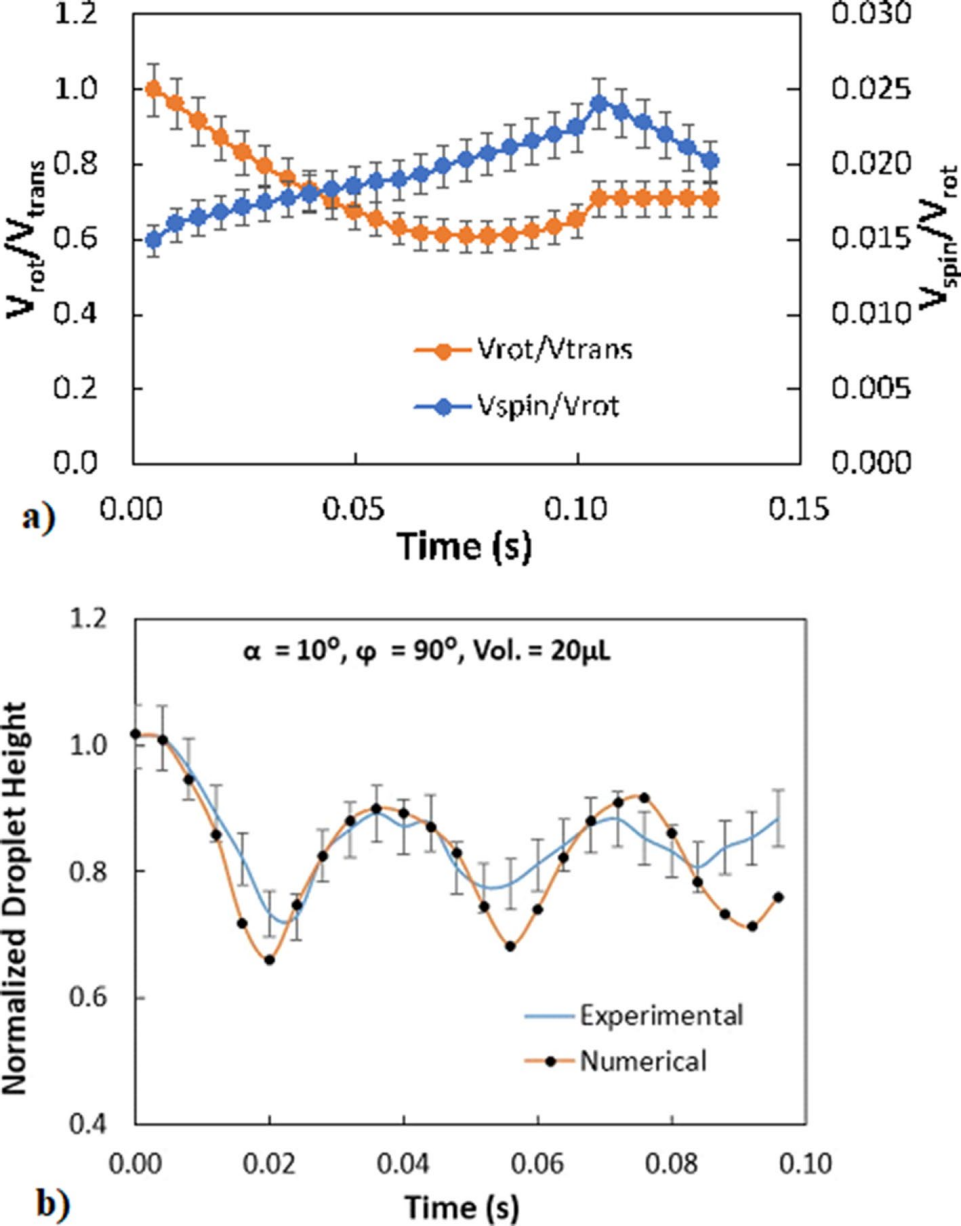
(**a**) Temporal behavior of velocity ratios for rotational and spinning velocities obtained from experiments, (**b**) Normalized maximum droplet height with time obtained from predictions and experiment. Droplet height is normalized by spherical droplet diameter of same volume.

The droplet rolls and spins because of imbalanced vertical and tangential forces in the inclined wedge structure having different hydrophobic plates (Fig. 1a). As the droplet rolls it puddles because of the gravitational influence. The maximum puddle height of the droplet can be approximated by $\sqrt{2(1-cos\theta )\frac{\gamma }{\rho g}}$, here, *θ* is contact angle and *ρ* is fluid density ^32^. The puddling modifies the mass center of droplet with time during rolling due to wobbling of the droplet. However, the droplet remains almost spherical as the droplet diameter becomes comparable to the capillary length (${\kappa }^{-1}=\sqrt{\frac{\gamma }{\rho g}}$, here ${\kappa }^{-1}$ is the capillarity length), i.e. the droplet rolls like a spherical marble without undergoing puddling. For the water droplet, the capillary length is in the order of 2.32 mm and the maximum volume of the droplet for marble-like rolling is about 6.53 μL, which is considerably small for the self-cleaning applications. This is because of the fact that large volume droplets give rise to a wide droplet width along the droplet path on the surface and a large number of dust particles can be removed by the droplet fluid within the droplet path, i.e. the dust removed area within the droplet path remains large. On the other hand, the large volume droplet wobbles during rolling, because of the puddling effect even though the wetted area on the surface remains large. Wobbling causes the change of the droplet center of mass during rolling. This results in a dynamically varying center of pressure in the droplet fluid, which further contributes to the wobbling. The tangential velocity of the rolling droplet (due to angular velocity) over the droplet translational velocity (ωR/V) can affect the wobbling because of the pressure variation in between the droplet liquid and its ambient air^33^. Hence, the inertial force differential created in droplet fluid and droplet ambient can be considered estimating the influence of the dynamic pressure change on the wobbling. This can be evaluated through the relation $\varphi =\frac{\Delta \rho {\omega }^{2}{R}^{2}}{{\rho }_{a}{V}^{2}}$, ^33^ here Δρ being density variation of droplet fluid and air surroundings and *ρ*_*a*_ is density of air, *ω*_*x*_ is the rolling angular velocity, *R* is the droplet radius, and *V* is the droplet translational velocity. For large values of φ (≫ 1), this effect becomes small on the wobbling. For droplet volumes 20–40 µL, values of $\varphi$ vary within between 800 and 950, which is greater than unity. Hence, the influence of dynamic change of center of pressure within the fluid does not significantly influence the droplet wobbling. Moreover, wobbling of the rolling droplet gives rise to potential energy change because of reorientation of the center of mass of the droplet. The potential energy difference between the droplet under puddling and the spherical droplet can be approximated as $\gamma \lambda$ ^2^ ≅ *ρgR*^*3*^, where λ is the difference between the center of mass due to puddle and spherical droplet^34^. The droplet wetting length is about $l\sim \sqrt{R\lambda }$ over the rolling surface and potential energy change of the spherical and puddled droplets having the same radius can be simplified as $\rho g{R}^{3}\lambda \sim \gamma {l}^{4}/{R}^{2}$.34 Hence, the wetting length of the puddled droplet can be approximated as $l\cong {R}^{2}/\sqrt{\frac{\gamma }{\rho g}}$, here $\sqrt{\frac{\gamma }{\rho g}}$ corresponds to the capillary length. The droplet center of mass shift ($\lambda$) becomes ~ *R*^*3*^ $/\frac{\gamma }{\rho g}$. For large volume droplets (20–40 μL), $\lambda$ yields large values (> 0.5 mm) and the difference between the center of mass of the puddled and spherical droplet becomes large. Therefore, the large volume rolling droplet gives rise to dynamically changing the wetting length over the hydrophobic surface, which creates a striation-like pattern of the wetted length over the rolled surface. The Bond number of the droplet is related to droplet gravitational force over the surface tension force and it can be expressed as: $\frac{\Delta \rho g{R}_{H}^{2}}{\gamma }$ here *R*_*H*_ is the hydraulic radius and *Δρ* is the density difference between the droplet fluid and the surrounding air. The Bond number is in the range of 2.69 × 10^–3^. It is worth mentioning that the droplet hydraulic diameter is almost half of the capillary length (2.73 × 10^–3^); hence, increasing droplet diameter enhances the Bond number so that droplet wobbling increases^9^. The droplet Weber number is associated with the relative importance of the droplet inertia force over the surface tension force. Since the droplet rolls and spins within the wedge, inertia force differs for rolling and spinning motions of the droplet. The Weber number for rolling (We_R_) takes the form $\frac{\rho {\omega }_{x}^{2}{R}_{H}}{\gamma }$ while spinning Weber number (We_s_) is $\frac{\rho {\omega }_{z}^{2}{R}_{H}}{\gamma }$, here ω_x_ and ω_z_ are the angular velocity of the droplet corresponding to rolling and spinning, respectively. The maximum rolling Webber number of the droplet is about 1.53 and the maximum spinning Weber number is about 0.051. Hence, the ratio of the rolling over the spinning Weber numbers (We_R_/We_s_) is about 30. This is similar magnitude of the ratio of droplet inertial forces due rolling over the spinning, i.e. droplet rolling dominates over the droplet spinning motion. Figure 7b shows temporal behavior of rolling/spinning droplet angular velocities obtained from the numerical predictions and the experiment. The wedge angle is 90° and the wedge inclination angle is 10°. The contact angle on the left surface of the wedge is 145° while it is 160° on the right wedge surface. The images of flow velocity inside the droplet obtained from the predictions are also shown in the figure. Rotational velocity remains larger for rolling than the spinning of the droplet. The predictions and experimental results for rotational velocities are in good agreement. However, the analytical predictions (Fig. 7a) of the early study ^9^ for angular velocity differ from the numerical and experimental findings. This is because, in the analytical solutions, the rolling and spinning droplet is kept in a spherical rigid geometry without going through the geometric deformation under the gravitational effect in the wedge. Hence, the oscillation in the rotational velocity curve is not observed unlike the numerical predictions and the experimental results. The oscillation in the rotational velocity is linked to the droplet wobbling in the wedge. In this case, as the droplet wobbles; i) the droplet wetting area (three-phase contact-line) over the hydrophobic wedge plate changes, and ii) advancing and receding angles of the rolling droplet also change. These alter the droplet adhesion over the hydrophobic surface while adversely affecting the droplet motion (rolling) within the wedge. As the size of the three-phase-contact length reduces during wobbling, adhesion force, due to the in-plane component of the surface tension force, reduces. This lowers the droplet rolling angular velocity within the wedge. Similar arguments are applicable for the droplet pinning. However, the oscillation in the spinning rotation velocity is relatively smaller than that of the rolling rotational velocity. Hence, the influence of the droplet adhesion over the hydrophobic wedge surfaces has considerable effect on the droplet rolling. Figure 8a shows the temporal behavior of the velocity ratio of rotational tangential velocity ($\omega R$) to the droplet translation velocity. The spinning velocity over the rotational velocity ratio is also shown in the Fig. 8a. It should be noted that the velocity data is obtained from the experiments. The droplet translational velocity is the vector sum of the tangential rotational velocity and the slipping velocity of the droplet over the surface. The slipping velocity is smaller than the tangential rotational velocity of the droplet^8^. As the time increases, the ratio of rotational velocity to translational velocity reduces while the ratio of spinning velocity to translational velocity increases. This indicates that as the droplet rolls down in the wedge, the droplet slip and spinning velocities increase. Figure 8b shows temporal behavior of the maximum normalized vertical height of the droplet in the wedge. The maximum vertical height of the droplet is obtained from the experiment and the simulations. The maximum vertical height of the droplet oscillates with time, which is similar to the droplet angular velocities as shown in Fig. 7b. Moreover, the oscillation in the maximum droplet height reduces with time while showing that rolling/spinning moments of the droplet suppresses the droplet geometric change in the wedge. Hence, as the droplet spinning enhances, the oscillatory behavior of the droplet in the geometric feature becomes less.

### Flow field inside droplet fluid

**Figure 9:**
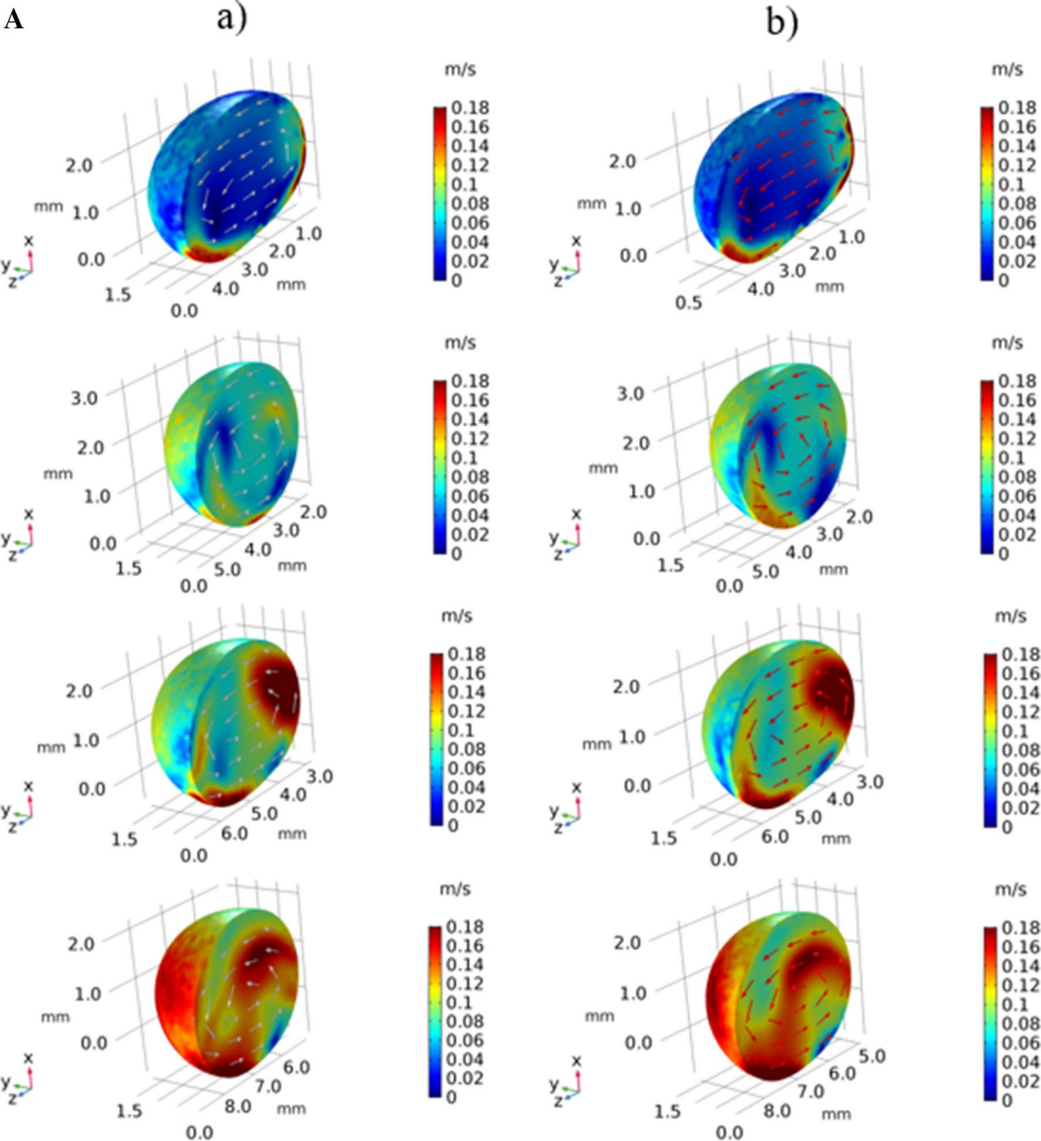

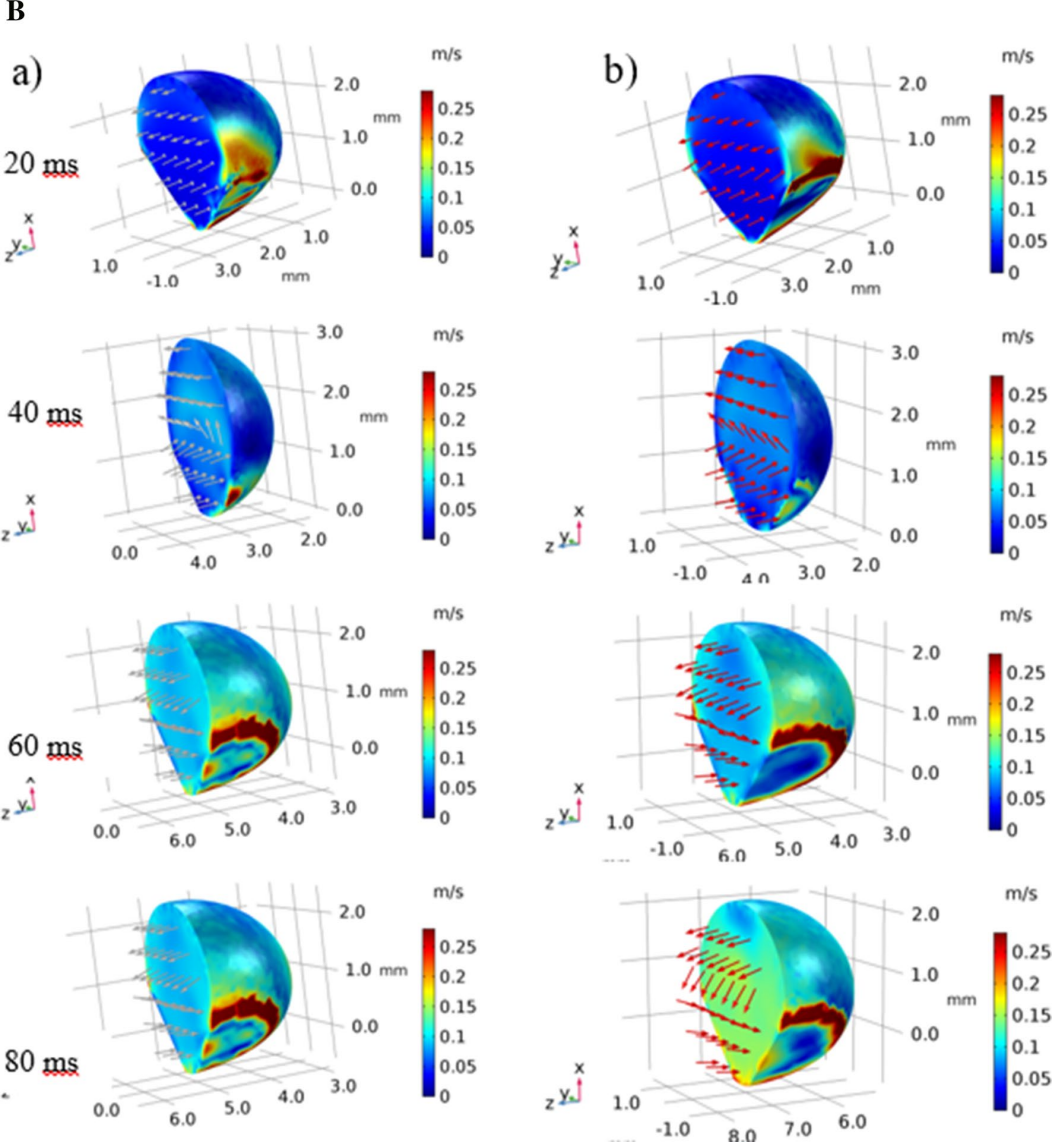
(**a**) Velocity contours inside droplet in inclined hydrophobic wedged for: (a) 145°-160° and (b) 150°-150°contact angles. (**b**) Pressure contours inside droplet in inclined hydrophobic wedged for: (a) 145°-160° and (b) 150°-150°contact angles.

**Figure 10:**
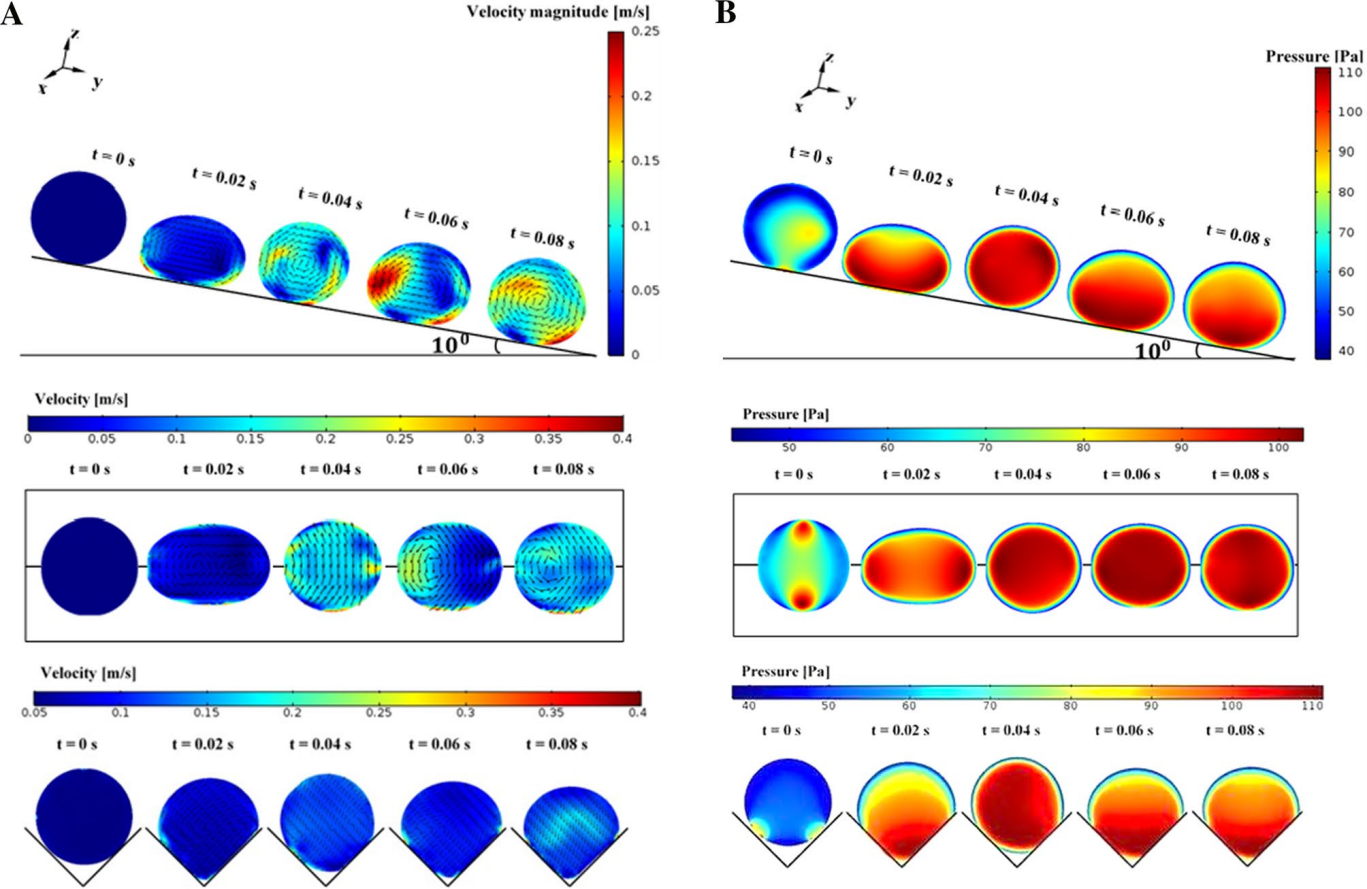
(**a**) Velocity contours across cross-sections for rolling and spinning droplet in hydrophobic wedge. (**b**) Pressure contours across cross-sections for rolling and spinning droplet in hydrophobic wedge.

The flow developed inside droplet is mainly influenced by the deformation of the droplet geometric feature under gravity, dynamic response of the droplet fluid under rolling and spinning motions, and interfacial shear between the droplet fluid and the hydrophobic wedge plates. Figure 9a,b show 3-dimensional views of the flow field and pressure distribution inside the droplet at different times in the wedge for two hydrophobic states of the wedge plates (contact angles of the plates 145°–160°). The spinning is initiated in the wedge because of differing hydrophobic wetting states of the wedge plates. This lowers the maximum velocity magnitude inside the droplet as compared to that corresponding to the droplet rolling over flat surface case. The maximum velocity becomes larger for rolling droplets within wedge having the plates of same hydrophobic wetting states. The attainment of the reduced maximum velocity for the spinning and rolling droplet is because of the tangential momentum gain of the droplet under spinning. In this case, the rolling momentum is reduced resulting in reduced droplet velocity in the rolling direction. Figure 10a,b show the velocity and pressure contours inside the droplet at different periods along the vertical (z-axis plane), horizontal (x-axis plane), and top (y-axis plane) planes for the wedge plates with different hydrophobic states (contact angles of 145° and 160°). In the case of the velocity field along the vertical plane (Fig. 10a), the droplet rotation creates unique circulation cell structures in the droplet fluid. The location of the maximum velocity changes as the rolling and spinning progress. The horizontal cross-section of the droplet reveals that flow creates a circulation cell within the spinning direction and the orientation of the circulation cell changes with progressing time. This demonstrates rates that the spinning axis changes as the droplet rolls in the wedge. In the case of the pressure contours (Fig. 10b), the high pressure region changes within the droplet fluid as the droplet spins and rolls. Pressure attains lower values as the flow velocity increases in the droplet fluid. Moreover, the pressure is within the range of 100 Pa (gage) in the droplet fluid and the pressure reduces towards the droplet free surfaces.

**Figure 11:**
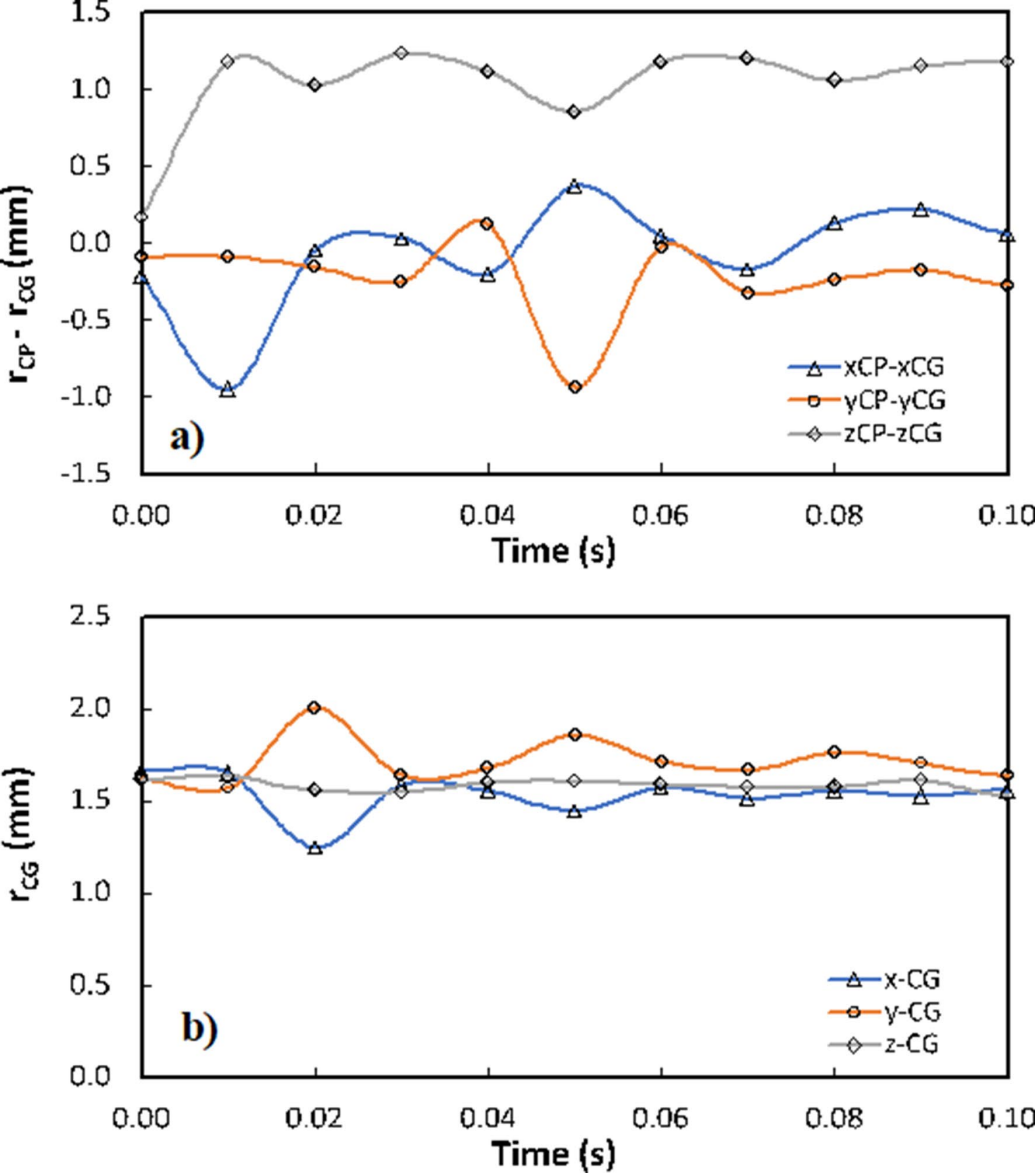
(**a**) Temporal variation of difference between center of pressure (r_cp_) and centroidal point (r_CG_) for rolling and spinning droplet in hydrophobic wedge, and (**b**) temporal variation of centroidal point (r_CG_) for rolling and spinning droplet in hydrophobic wedge. x_cp_, y_cp_, z_cp_, and x_CG_, y_CG_, z_CG_ represents the center of pressure and centroidal point from x,y, and x-axes.

On the other hand, in the interfacial region of the contact between the droplet fluid and solid, the velocity field is affected by the interfacial shear developed in the droplet fluid. The wetted surface is hydrophobic and the slip condition results in slip velocity at the interface. Moreover, during the rotation and spinning, the location of the droplet centroidal point changes because of droplet wobbling. In addition, the center of pressure inside the droplet fluid also changes. Figure 11a shows the distance between the center of pressure and the centroidal point inside the droplet with time while Fig. 11b shows the location of the centroidal point with time. Since the droplet motion does not possess the axisymmetric behavior during rolling and spinning in the wedge, the distance between the center of pressure and the centroidal point becomes different along x, y, and z-axes. Hence, the center of pressure can be determined from the expression: ${\chi }_{cp}=\frac{{\int }_{A}{\chi }_{i}Pd{A}_{i}}{{\int }_{A}Pd{A}_{i}}$, where *xi* is the distance from the horizon point of the droplet (droplet free surface) along x, y, and z-axes and index *i* represents the x, y, z-axes, *P* is the pressure and *Ai* is the cross-sectional area of the droplet normal to each axis. The difference between the centroidal point and the center of pressure becomes large along the x-axis (horizontal axis, Fig. 11a). This is because the droplet x-axis is normal to the vertical axis (gravitational axis, i.e. z-axis) and the droplet wets the hydrophobic plates in the wedge along the x-axis. Consequently, during the droplet wobbling centroidal point changes while causing large change along the x-axis. In the case of y and z-axes (Fig. 11b), the change of centroidal point shows oscillations because of the droplet wobbling; however, this variation is relatively smaller as compared to that of the x-axis. Consequently, the effect of interfacial shear becomes important on the flow developed in the droplet, particularly along the y and x-axis, i.e. the shear rate becomes small because of large values of wetting length (*l*m) in ${\mu }_{w}(\frac{{V}_{n}-{u}_{s}}{{l}_{m}})$. In addition, the inertia force over the gravitational force increases with progressing time, which gives rise to increased Weber and Bond numbers, i.e. increase in ω*x* and ω*z* enhances the Weber numbers for rolling ($\frac{\rho {\omega }_{x}^{2}{R}_{H}}{\gamma }$) and spinning ( $\frac{\rho {\omega }_{z}^{2}{R}_{H}}{\gamma }$). Hence, the fluid inertial forces have a considerable effect on the flow field as the rolling and spinning progresses. The Bond number ($\frac{\Delta \rho g{R}_{H}^{2}}{\gamma }$) mainly influenced by the droplet hydraulic radius (*RH*) during rolling and spinning. However, the change of the hydraulic radius with time remains small. Nevertheless, the influence of the weight force on the flow field becomes important as the center of mass alters with time inside the droplet. Consequently, the shear force created in the droplet fluid at the interfacial contact and the flow forces generated under the gravitational influence (because of the change of center of gravity during rolling and spinning) result in a complicated flow field in three-dimensional space inside the droplet fluid (Fig. 9a).

**Figure 12:**
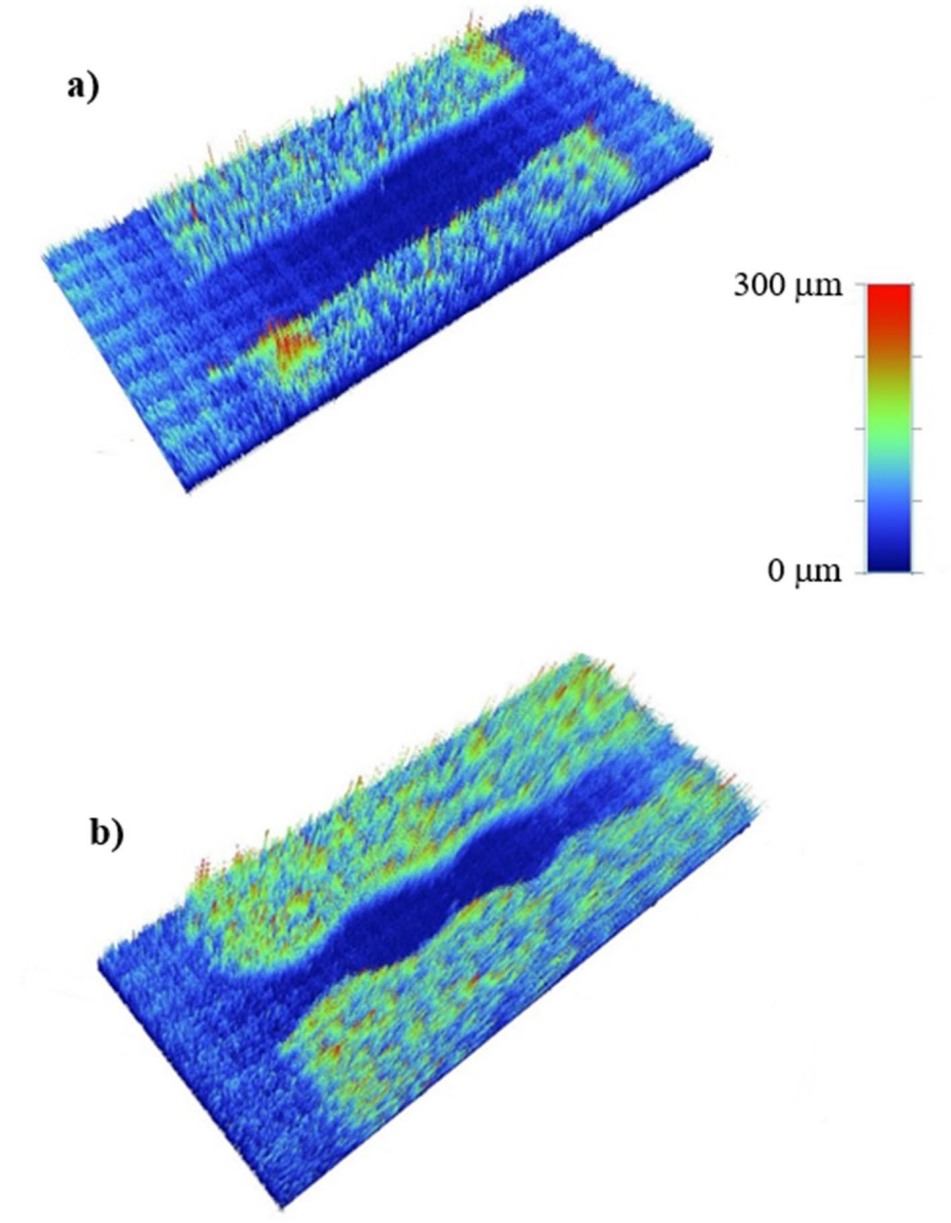
Droplet path on dusty hydrophobic plain surface: (**a**) rolling and spinning droplet, and (**b**) rolling only droplet.

To evaluate the effect of droplet spinning and rolling on the droplet path over the flat hydrophobic surface, further tests are conducted. In this case, the dust particles (about 150 μm uniform thickness) are deposited over the horizontally located hydrophobic plain surface. Later, the wedge fixture is located behind the horizontally situated dusty hydrophobic surface so that the rolling and spinning droplet leaving the wedge continues its motion on the dusty hydrophobic surface. The path of the spinning and rolling droplet path on the dusty hydrophobic surface is monitored by using high-speed optical cameras. Similarly, the path of the rolling only droplet (not spinning) on the dusty hydrophobic surface is also monitored by using the optical high-speed camera for comparison. Figure 12 shows the three-dimensional optical images of the droplet paths corresponding to spinning and rolling, and rolling only cases. It is evident that the edges of the droplet path remain more straight for the spinning and rolling droplet as compared to that of rolling only droplet. The ratio of area (where the dust is removed within the droplet path) due to rolling droplet only to rolling and spinning droplet cases is about 60%. Hence, the dust mitigated area by rolling and spinning droplet becomes notably larger than that of the rolling droplet only case.

## Conclusion

Droplet rolling and spinning behavior is examined in the inclined wedge, which is formed from two hydrophobic plates with different wetting states. Hydrophobized surfaces have contact angles 160° ± 2° and 140° ± 2° with a hysteresis of 5° ± 1°. Since the wedge is inclined from the horizontal plane (normal to gravitational direction), the water droplet rolls under the gravity and the tangential momentum, which is developed because of different pinning forces on the hydrophobized plate surfaces, results in droplet spinning during the rolling. Depending on the relative magnitude of rolling and tangential momentum, the spinning axis can change during rolling. To spin the droplet along its symmetry axis, the droplet's tangential momentum needs to maintain constant during the rolling. Hence, the fixture is located precisely and the uniformity of the wetting states on the hydrophobized surfaces is ensured. The rolling and spinning droplet path on the horizontally located hydrophobic plane surface is assessed depositing the dust particles over the surface. The resulting droplet path, along which the dust particles are removed, is evaluated and compared to that of the path created by the rolling only droplet. The droplet motion and flow field inside the droplet are monitored using the high-speed recording facility. In addition, the flow field inside the droplet is simulated in 3D geometry resembling the actual droplet behavior in the wedge fixture. The flow field predicted is validated with those obtained from the experiments. The findings reveal that the velocity predictions agree well with its counterparts obtained from the experiments. The local adhesion of the rolling droplet on the hydrophobic surfaces in the wedge (through a three-phase-contact line) causes the complicated flow field developing inside the droplet fluid. The shear influence at the droplet-wedge interface adds to the flow complication inside the droplet fluid. In this case, localized circulation cells in the horizontal plane (across-the wetted hydrophobic surfaces) are created. The droplet wobbling during rolling (under the gravitational influence) alters the size and location of the circulation cells. In this case, change of the center of pressure and centroidal point of the droplet causes droplet momentum change, which in turn, alters the orientation of the spinning axis. However, the change of the spinning axis is found to be small. The difference between the droplet translational and tangential velocities indicates that the droplet slips along the wedge plates during rolling and spinning. The droplet slipping is link to the slip-length of the hydrophobic surface, which is found to be small and the slip velocity becomes considerably smaller than those of rolling and spinning velocities. The rolling velocity of the droplet remains larger than the spinning velocity. The droplet path has parallel-sided edges on the dusty hydrophobic plate surface for the case of spinning and rolling droplets. The rolling only droplet results in striation like edges of the droplet path over the dusty hydrophobic surface. Hence, the spinning of the rolling droplet improves the area cleaned over the dusty hydrophobic surface. The present study gives a detailed analysis of the flow field created by the rolling and spinning droplet fluid in the inclined hydrophobic wedge. It delivers valuable information for the dust mitigation ability of spinning and rolling droplets over the hydrophobic surfaces.

## Supplementary Information

Supplementary Information.
