# Supplementary material for: On the mechanism of droplet rolling and spinning in inclined hydrophobic plates in wedge with different wetting states
Source: Sci Rep. 2021 Jul 23;11:15086. doi: 10.1038/s41598-021-94523-8 (PMC8302624; doi:10.1038/s41598-021-94523-8)
Supplement: Supplementary file 1 — Supplementary Information. [file 41598_2021_94523_MOESM1_ESM.docx]

**Supporting Information**

**ON THE MECHANISM OF DROPLET ROLLING AND SPINNING IN INCLINED HYDROPHOBIC PLATES IN WEDGE WITH DIFFERENT WETTING STATES**

Bekir Sami Yilbas^1,2,3^ Mubarak Yakubu^1^ Abba Abdulhamid Abubakar^1^ Hussain Al-Qahtani^1^ Ahmet Sahin^1^ Abdullah Al-Sharafi^1,2,3^

^1^Mechanical Engineering Department, King Fahd University of Petroleum and Minerals, Dhahran, 31261, Saudi Arabia

^2^Center of Research Excellence in Renewable Energy (CoRE-RE), KFUPM, Dhahran, 31261, Saudi Arabia

^3^Senior Researcher at K.A. CARE Energy Research & Innovation Center at Dhahran, Saudi Arabia

Correspondence and requests for materials should be addressed to B.S.Y. (Email: bsyilbas@kfupm.edu.sa; Phone: +966 3 860 4481)

**S1: Droplet Rolling and Spinning**

The momentum balance for the rolling droplet results in angular velocity (*ω_x_*) in the wedge fixture form of:^1^

$\omega_{x}=\frac{gt^{2} sin \alpha}{R\sin\frac{\phi}{2}\left( 1+\frac{2}{5{sin}^{2} \left( \frac{\phi}{2} \right)} \right)}$ (S1)

where *g* is the gravitational acceleration, *t* is the time, and *α* is the inclination angle of the wedge along the horizontal plane), *R* represents droplet hydraulic diameter, $\phi$ is wedge angle of the fixture.

However, droplet spinning is resulted because of pinning forces in balance in the transverse direction. The mathematical arrangement for the spherical droplet spinning is given in the early study.^1^ The droplet spinning can be expressed in:

 (S2)

Here, *A* is:

$A=\frac{a^{2}}{R^{2}\sin^{2} \left( \frac{\phi}{2} \right)}$ (S3)

and *K* is:

$K=\frac{120\gamma\left[ f_{1}\left( \cos\theta_{R1}-\cos\theta_{A1})-f_{2}(\cos\theta_{R2}-\cos\theta_{A2} \right) \right]}{\rho\pi^{3}\forall\cos(\frac{\phi}{2})}$ (S4)

Reducing the droplet hydraulic radius while increasing linear acceleration increases the value of A. In addition, reducing droplet volume and increasing the retention force differences between two hydrophobic surfaces ($f_{1}\left( \cos\theta_{R1}-\cos\theta_{A1})-f_{2}(\cos\theta_{R2}-\cos\theta_{A2} \right)$), the value of K becomes large. Increasing the wedge angle of the fixture (*φ*), lowers the term $\frac{\left( 1+\frac{2}{5{sin}^{2} \left( \frac{\phi}{2} \right)} \right)^{2}}{R^{2}{sin}^{2} \left( \frac{\phi}{2} \right)}$.

where, *a* is the linear acceleration (*a* $=\frac{g\sin\alpha}{1+\frac{2}{5\sin^{2} \left( \frac{\phi}{2} \right)}}$, here $\theta_{R1}$, $\theta_{A1}$, $\theta_{R2}, \mathrm{and} \theta_{A2}$ are advancing and receding angles of the rolling/spinning droplet on surfaces 1 (right hand side surface) and 2 (left hand side plate surface), respectively, *f_1_* and *f_2_* are the solid fractions of hydrophobic surfaces 1 and 2, respectively, $\forall$ is droplet volume, and $\rho$ is the density of droplet fluid. To spin the droplet in the wedge of two hydrophobic surfaces with different wetting states, the following is to be satisfied:

$t^{2}\left( {A+K}^{2} \right)-\frac{\left( 1+\frac{2}{5{sin}^{2} \left( \frac{\phi}{2} \right)} \right)^{2}}{R^{2}{sin}^{2} \left( \frac{\phi}{2} \right)}$ (S5)

In this case, both *A* and *K* remain large since the time (*t*) is in order of the fraction of the second. The value of *A* depends on the linear acceleration (*a*) of the droplet, inclination angle of the wedge (*α*), the wedge angle (*φ*), and hydraulic radius of the droplet. Moreover, the spinning axis angle (*θ_s_*), can be written as:^1^

$\theta_{s}={sin}^{-1}\left( \frac{gt sin \alpha}{Rt\sqrt{\left( {A+K}^{2} \right)}\sin\frac{\phi}{2}\left( 1+\frac{2}{5{sin}^{2} \left( \frac{\phi}{2} \right)} \right)} \right)$ (S6)

Since the spinning angle is measured from the vertical direction, the spinning axis becomes normal to the horizontal plane where the droplet rolls, i.e. the spinning axis becomes normal to the horizontal plane of the wedge.

**S2: Droplet Pinning and Interfacial Resistance**

The retention force created during droplet rolling over the hydrophobic surface, due to advancing and receding angles of the droplet, can be written as:^2^ $F_{ad}=\frac{24}{\pi^{3}}\gamma fD_{w}(cos\theta_{R}-cos\theta_{A})$ here, *γ* is surface tension of droplet fluid, *f* being solid fraction (solid-liquid contact fraction because of the surface texture, i.e. the ratio of the area of pillars over the area projected on the surface), *D_w_* is droplet wetted diameter, $\theta_{R}$ and $\theta_{A}$ are receding and advancing angles of droplet on the rolling surface. It is worth to note that the droplet adhesion force is associated with the $F_{ad}=\frac{24}{\pi^{3}}\gamma fD(cos\theta_{R}-cos\theta_{A})$, here *D_w_* is droplet wetted diameter on the wedge side plate surface, $\theta_{R}$ and $\theta_{A}$ are the droplet receding and advancing angles on the same surface.

The interfacial velocity on the hydrophobic surface is related to the slip length, which takes the form: $\frac{b}{L}=\frac{A}{r^{2}}-B$ here *A* and B being the constant, *r* represents the solid fraction of the surface while *L* being the length in between two consecutive pillars in the texture.^3,4^ The solid fraction corresponds to the area of pillars over the projected area of the texture. The effective slip length (*b*) can be approximated by $b_{eff}=L\left( \frac{0.325}{\sqrt{r}}-0.44 \right)$.^5^ In line with the AFM line scan (Figure 6c), the solid fraction of the hydrophobized surface is in the order of 0.47 and L is about 100 nm. This results in an effective slip length of 3.41 nm. Moreover, the interfacial slip velocity can be expressed in the form of $u_{s}=\frac{b_{eff}}{\mu}\tau_{wi}$, here $\tau_{wi}$ is the interfacial shear stress.^6^ In line with the analogy for the Couette flow, the slip velocity (*u_s_*) takes the form $u_{s}\sim\frac{b_{eff}}{h_{t}}u_{f}$, here *u_f_* is the flow velocity on the surface. It is worth to mention that the flow velocity at interface is similar order of the velocity magnitude in the droplet fluid. Hence, the shear stress formed in the droplet fluid because of droplet rolling and spinning, and the slip velocity at the interface is linked to the shear rate. The shear stress can be approximated as $\sim\mu_{w}(\frac{V_{n}-u_{s}}{l_{m}})$, here $V_{n}=\sqrt{V_{r}^{2}+V_{s}^{2}} sin \delta$ (where *V_r_* and *V_s_* are the tangential velocities of rotation and spinning, and *δ* is the inclination angle of the surface), *V_n_* is the component of the velocity magnitude normal to the solid surface *l_m_* is the distance from solid surface to the droplet centroidal point.

**References**

1. Yakubu, M., Yilbas, B. S., Abubakr, A. A. & Al-Qahtani, H. Droplet Rolling and Spinning in V-Shaped Hydrophobic Surfaces for Environmental Dust Mitigation. *Molecules* **25**, 3039 (2020).

2. Yilbas, B. S., Al-Sharafi, A., Ali, H. & Al-Aqeeli, N. Dynamics of a water droplet on a hydrophobic inclined surface: influence of droplet size and surface inclination angle on droplet rolling. *Rsc Adv.* **7**, 48806–48818 (2017).

3. Ybert, C., Barentin, C., Cottin-Bizonne, C., Joseph, P. & Bocquet, L. Achieving large slip with superhydrophobic surfaces: Scaling laws for generic geometries. *Phys. fluids* **19**, 123601 (2007).

4. Ng, C.-O. & Wang, C. Y. Apparent slip arising from Stokes shear flow over a bidimensional patterned surface. *Microfluid. Nanofluidics* **8**, 361–371 (2010).

5. Maali, A. & Bhushan, B. Measurement of slip length on superhydrophobic surfaces. *Philos. Trans. R. Soc. A Math. Phys. Eng. Sci.* **370**, 2304–2320 (2012).

6. Smith, J. D. *et al.* Droplet mobility on lubricant-impregnated surfaces. *Soft Matter* **9**, 1772–1780 (2013).
